# Improvement of electrocardiographic diagnostic accuracy of left ventricular hypertrophy using a Machine Learning approach

**DOI:** 10.1371/journal.pone.0232657

**Published:** 2020-05-13

**Authors:** Fernando De la Garza-Salazar, Maria Elena Romero-Ibarguengoitia, Elias Abraham Rodriguez-Diaz, Jose Ramón Azpiri-Lopez, Arnulfo González-Cantu

**Affiliations:** 1 Universidad de Monterrey, Escuela de Medicina, Especialidades Médicas, Monterrey, Nuevo León, Mexico; 2 Departamento de Medicina Interna, Hospital Christus Muguerza Alta Especialidad, Monterrey, Nuevo Leon, Mexico; 3 Direccion de Enseñanza e Investigación en Salud, Hospital Christus Muguerza, Alta Especialdiad, Monterrey, Nuevo León, México; 4 Departamento de Cardiología, Hospital Christus Muguerza, Alta Especialidad, Monterrey, Nuevo León, México; Universiti Tun Hussein Onn Malaysia, MALAYSIA

## Abstract

The electrocardiogram (ECG) is the most common tool used to predict left ventricular hypertrophy (LVH). However, it is limited by its low accuracy (<60%) and sensitivity (30%). We set forth the hypothesis that the Machine Learning (ML) *C5*.*0 algorithm* could optimize the ECG in the prediction of LVH by echocardiography (Echo) while also establishing ECG-LVH phenotypes. We used Echo as the standard diagnostic tool to detect LVH and measured the ECG abnormalities found in Echo-LVH. We included 432 patients (power = 99%). Of these, 202 patients (46.7%) had Echo-LVH and 240 (55.6%) were males. We included a wide range of ventricular masses and Echo-LVH severities which were classified as mild (n = 77, 38.1%), moderate (n = 50, 24.7%) and severe (n = 75, 37.1%). Data was divided into a training/testing set (80%/20%) and we applied logistic regression analysis on the ECG measurements. The logistic regression model with the best ability to identify Echo-LVH was introduced into the *C5*.*0* ML algorithm. We created multiple decision trees and selected the tree with the highest performance. The resultant five-level binary decision tree used only six predictive variables and had an accuracy of 71.4% (95%CI, 65.5–80.2), a sensitivity of 79.6%, specificity of 53%, positive predictive value of 66.6% and a negative predictive value of 69.3%. Internal validation reached a mean accuracy of 71.4% (64.4–78.5). Our results were reproduced in a second validation group and a similar diagnostic accuracy was obtained, 73.3% (95%CI, 65.5–80.2), sensitivity (81.6%), specificity (69.3%), positive predictive value (56.3%) and negative predictive value (88.6%). We calculated the Romhilt-Estes multilevel score and compared it to our model. The accuracy of the Romhilt-Estes system had an accuracy of 61.3% (CI95%, 56.5–65.9), a sensitivity of 23.2% and a specificity of 94.8% with similar results in the external validation group. In conclusion, the *C5*.*0* ML algorithm surpassed the accuracy of current ECG criteria in the detection of Echo-LVH. Our new criteria hinge on ECG abnormalities that identify high-risk patients and provide some insight on electrogenesis in Echo-LVH.

## Introduction

Since 1909, over thirty-six electrocardiographic left ventricular hypertrophy (ECG-LVH) criteria have been proposed, but most are redundant or oversimplify the electrical changes in LVH [1, 2]. Most criteria (i.e. Cornell, Sokolov-Lyon) are based solely on increased QRS voltage, but this is not a consistent finding in all patients with ECG-LVH [1, 3, 4]. A more realistic approach was developed by Romhilt-Estes in 1968, when they created a multilevel score system using a logistic regression model based on a broad spectrum of ECG abnormalities associated with ECG-LVH (i.e. QRS voltage, ST “strain” pattern, QRS duration), although its sensitivity (≈30%) and accuracy (≈60%) are low [5, 6]. Additionally, in the 21^st^ century almost everyone agrees that the ECG´s role in Echo-LVH should also provide a basic understanding of the electrical remodelsing inherent to hypertrophy [7]. New statistical and computational algorithm modeling is needed in order to evaluate ECG patterns that could predict LVH more accurately. A Machine Learning (ML) approach could be useful in these cases.

ML, a subset of artificial intelligence, is defined as the ability of a system to autonomously acquire knowledge via the extraction of patterns from large databases [8]. Several domains of ML have been applied in ECG in order to improve LVH detection capability, but some are considered “*black boxes”* so the clinician is unable to determine why a certain patient is classified as having LVH, or these studies may be too complex to use in daily clinical practice [9]. One ML domain that surpasses these two limitations is the *C5*.*0 algorithm*, which generates a multilevel binary tree using ECG *features* that most contribute to the classification of patients as Echo-LVH in an easy to understand manner [10].

We used the ML *C5*.*0 algorithm* to optimize the ECG in the detection of Echo-LVH while also generating insights on the electrical phenotypes of the hypertrophied myocardium by creating a comprehensive and clinician-friendly multilevel binary decision tree.

## Materials and methods

This study followed STARD methodology [11] and international guidelines for the development of ML models [12]. This study complies with the Declaration of Helsinki, and our local ethics committee (Grupo Christus Muguerza, approval number CMHAE-001-19) approved the research protocol. Precaution was taken to protect the privacy and confidentiality of the research subjects; all data was anonymized. Since it was a retrospective study, informed consent was waived.

### Study design

This was an observational, retrospective case-control study that included data from a representative sample of consecutive adult patients who underwent an echocardiogram (Echo) and an ECG between January 2016 and June 2018. The study was conducted in the Cardiology Department of the *Hospital Christus Muguerza Alta Especialidad* in Monterrey, Mexico.

#### Eligibility criteria

We included men and women over 35 years of age–as recommended by the 2009 American ECG guidelines—who underwent a transthoracic Echo and a 12-lead ECG during the same hospital admission. The anthropometric data and medical history of the population were obtained from the medical charts and included age, gender, weight (kg), height (cm) and relevant medical background (i.e. hypertension, type 2 diabetes mellitus, congestive heart failure). The body mass index (BMI) was reported in kg/m^2^; the body surface area (BSA) was obtained as follows and was reported in m^2^:

$$\sqrt{\left[\left(weight\;\times \;height\right)÷3600\right]}$$

Ischemic heart disease (IHD) is a highly prevalent disease in our population; in order to generalize our results, we included a subgroup of patients with subendocardial or transmural ischemia. For this purpose, IHD was defined as echocardiographic segmental hypokinesia or akinesia of a vascularized territory with or without pathological Q waves in 2 or more continuous leads. None of these patients had acute ischemic findings on ECG or acute ischemic syndrome.

The exclusion criteria were: preexcitation syndromes such as Wolff–Parkinson–White, acute ischemic findings in ECG, acute ischemic syndrome, elevated cardiac enzymes, tachycardia (>110 bpm), intraventricular conduction delays (left and/or right bundle branch block, left anterior and/or posterior fascicular block), pacemaker rhythms, fusion rhythms, patients who had undergone cardiotomy in the prior 3 months, hypertrophic cardiomyopathy (unexplained LVH, defined by increased wall thickness in 1 or more LV segments), dilated cardiomyopathy, interventricular septal defects, intensive care unit critically ill patients and those with incomplete anthropometric measurements.

#### Electrocardiography

We obtained a 12–lead ECG using Phillips “Pagewrite TC50” equipment (Best, Netherlands). All ECG were performed with a 25mm/sec velocity and a 10 mm/mV sensitivity. An Internal Medicine and Cardiology trainee measured the electrocardiographic variables (inter-observer kappa = 0.91 and intra-observer kappa = 0.96) using a *Phillips* graded scale “*TraceMasterVue”* software. We included the most frequent previously reported measurements pertaining to LVH [1], such as: S-wave voltage and R-wave voltage in all ECG leads (I, II, III, aVL, aVF, aVR and V1-V6), P-wave duration and voltage in the V1 lead, left atrial enlargement (LAE) defined as a negative deflection in lead V1 greater than one *Ashman unit* (40 ms x 0.1 mV), QRS complex duration in lead V1, QRS axis (using leads I and aVL), intrinsicoid deflection in lead V6 (qR duration ≥ 0.05 sec) and “ST strain" (downward ST depression >1 mm at 40ms from the J point with a downward slope, and asymmetric T wave inversion). Because of the low prevalence of the ST “strain” pattern in the classic definition of LAE, we decided to define it as [13]: 1) ST flat depression ≥1mm at 40ms of the J point with or without T wave inversion in V6, as defined by Minnesota’s code (MC 4–1), and 2) if the P wave´s negative component duration in lead V1 was greater than the initial positive component.

We calculated the Romhilt-Estes multilevel score as follows: R or S wave in any limb lead ≥2 mv, or S wave in V1 or V2 ≥3 mv, or R wave in V5 or V6 ≥3 mv (3 points); P negative terminal force equal or greater than one *Ashman unit* (3 points); ST “strain" pattern = downward ST depression >1 mm at 40ms from the J point with downward slope and with asymmetric T wave inversion, without digitalis (3 points); left axis deviation defined as QRS axis ≤ −30 degrees [2 points]; QRS duration ≥ 0.09 msec [1 point]; intrinsicoid deflection in V5 or V6 ≥ 0.05 msec [1 point], and scored LVH as ≥ 4 points [5].

#### Echocardiography

Three-licensed cardiologists performed a transthoracic Echo using the *“EPIQ7”* and “IE33” Phillips (Best, Netherlands) equipment (agreement kappa = 0.98). Measurements were made following the “*American Society of Echocardiography and the European Association of Cardiovascular Imaging”* recommendations [14]. In order to obtain the required measurements, we used a two-dimensional ECG-guided M mode approach. The following measurements were obtained: interventricular septum thickness in diastole (IVSTd), left ventricular internal diameter in diastole (LVIDd), left ventricular posterior wall thickness in diastole (LVPWTd), left ventricular mass (LVM, gr), left ventricular mass index (LMVI, gr/m^2^) and relative wall thickness (RWT). The formula used to calculate the LVM was the following [14]:

$$LVM=0.8\times \left\{1.04\left[{\left(LVIDd\;+LVPWTd\;+IVSTd\right)}^{3}-{\left(LVIDd\right)}^{3}\right]\right\}+0.6g$$

Indexation of the LVM was obtained using the BSA as recommended by the “*American Society of Echocardiography and the European Association of Cardiovascular Imaging”*. The following formula was used [14]:

LVMI=LVM÷BSA

LVH was defined as: male and female patients with a LVMI above 115 gr/m^2^ and 95 gr/m^2^, respectively [14]. Severity was classified as mild (116–131 gr/m^2^, 96–108 gr/m^2^) moderate (132–148 gr/m^2^, 109–121 gr/m^2^) and severe (>148 gr/m^2^, >121 gr/m^2^), in males and females, respectively [14]. The RWT was calculated using the following formula:

RWT=(2×LVPWTd)÷LVIDd

Different left ventricular morphologies were defined as: cardiac remodeling (normal LVMI with RWT >0.42), concentric hypertrophy (elevated LVMI with RWT >0.42) and eccentric hypertrophy (elevated LVMI with RWT ≤0.42) [14].

### Statistical analysis

For continuous variables, normality was established by computing skewness and kurtosis and by applying the Shapiro-Wilk test; log_10_ transformations were conducted when appropriate. Continuous variables were expressed as mean and standard deviation or confidence intervals, while categorical variables were expressed in frequencies and percentages. We used the two-sample t-test and Fisher´s exact test for group comparisons. The models were two-sided and the significant p- value was <0.05.

Data was divided into a training/testing set (80/20%) followed by logistic regression analysis using the forward stepwise method on ECG measurements. The set of independent variables with the best ability to classify the patients (lesser *Akaike Information Criteria or AIC*) were introduced into the classifying model.

We used the *C5*.*0* supervised ML algorithm to create a multilevel binary decision tree, using the ECG *features* that provided the greatest information to classify patients as having Echo-LVH [10]. The *feature* and cut-off value that contributed the most in the Echo-LVH classification, initially split the sample, thus creating two new sets of data (one for each partition branch). This process continued until a stop criterion was reached (i.e. all data was classified). This type of algorithm can take in account parameters in order to maximize its performance such as: a matrix cost that can be associated with possible errors (penalize misclassification, i.e. False positives a negative classification), or a subset of data in order to evaluate discrete predictors.

The last nodes are called “leaves” and contain the classification probabilities for each patient of having Echo-LVH; if it was greater than 0.5 (50%), the patient was classified as having LVH and *vice versa*. Decision tree models are known to over adjust, and this could compromise its generalization to new data. To avoid this, we pruned the tree, reaching a simple and exact decision tree. The algorithm did automatically the pruning process, by removing parts of the tree that are predicted to have a relative high error rate. This process was applied to every sub-tree.

In the process of modeling we first reduced the dimensionality of the data with a logistic regression and we maintained in the further steps the variables with highest estimates. These subsets of variables were included in the algorithm to get the classification tree. This step was replicated until we had a tree with biological coherence with myocardial hypertrophy (established by a cardiology expert J.R.A) and holding the principle of parsimony, to obtain a useful and practical tree. In order to improve the accuracy of the final tree, matrix costs were included in the algorithm (penalizing the false negatives), but we did not find a matrix cost that could improve accuracy, so the final tree was obtained from the default parameters of the algorithm.

Accuracy and confidence intervals, sensitivity, specificity, positive predictive value (PPV), and negative predictive value (NPV) of several decision trees were calculated. We selected the tree (in sort of clinical relevance) with the greatest accuracy, sensitivity and specificity, in order to have equal capabilities to detect positive Echo-LVH from negative Echo-LVH. We selected the combination of the three first ECG features according to the final decision tree and clinician discretion and calculated its diagnostic performance.

We calculated the same diagnostic parameters in the Romhilt-Estes model since it is the most frequently used multilevel score system and we compared them to our model.

No missing values in demographic, Echo and ECG parameters were accepted. Missing values in terms of comorbidities were accepted and were approached with complete case analyses. Sample size was calculated on the basis of a 40% sensitivity of reported conventional electrocardiographic criteria, with a delta of 0.1 (inferiority sensitivity limit = 30%) [1]. To reach 80% power and an alpha error <0.05, we required at least 155 patients in each group.

#### Internal validation

Data was divided into a training/testing set (80/20%) for internal validation.

#### External validation

The final model was validated in a second group of 150 new patients (47.3% cases) that were recruited between July 2018 and March 2019. Validation data corresponded to 34.2% of the initial sample. Accuracy and confidence intervals, sensitivity, specificity, PPV and NPV were calculated.

We used the statistics program SPSS *vs*. *24* and *R-studio vs 3*.*4*.*0*.

## Results

### Demographic and echocardiographic characteristics

The cardiology department conducted 4882 echocardiograms in the first study period; 1881 patients were eliminated because they were under 35 years of age. Incomplete Echo measurements were detected in 608 patients, and 1961 patients were eliminated when applying the remaining exclusion criteria. We included 154 patients with Echo-LVH and 230 controls. The Echo positive ischemic subgroup included 48 Echo-LVH. With a total of 432 patients, we reached a power of 99%. Males were more prevalent in the study than females (n = 240, 55.6%), and slightly more prevalent in the control group, 59% vs. 51% (p = 0.03). This difference was relevant in the subgroup of patients with hypokinesia or akinesia by Echo in comparison with patients who were negative for this finding (*p = 0*.*001 vs p = 0*.*580*). The mean (SD) age was 67.3 (17) years. **Table 1**shows a comparison between demographic, anthropometric and Echo measurements between both groups.

**Table 1:** Demographic and echocardiographic measurements of the population.

Mean (SD)	Total sample (n = 432)	UCG		p-value
		Negative LVH (n = 230)	Positive LVH (n = 202)	
**Demographic and Anthropometric**				
Age (years)	67.3 (13.7)	65.7 (14.8)	69.3 (12.1)	0.03
Weight (kg)	78.2 (16.7)	78.2 (16)	78.1 (17.4)	0.93
Height (cm)	167.4 (9.7)	168.3 (9.7)	166.2 (9.6)	0.04
BMI (kg/m^2^)	27.8 (4.9)	27.4 (4.3)	28.2 (5.4)	0.13
BSA (m^2^)	1.9 (0.24)	1.9 (0.23)	1.9 (0.24)	0.49
**UCG parameters**				
IVSTd	1.18 (0.27)	1.04 (0.19)	1.34 (0.26)	0.001
LVIDd	4.57 (0.75)	4.4 (0.64)	4.76 (0.8)	0.001
LVPWTd	1.18 (0.26)	1.04 (0.19)	1.32 (0.24)	0.001
RWT	0.54 (0.19)	0.49 (0.16)	0.58 (0.2)	0.001
LVM (gr)	201.3 (72.4)	157.4 (37.5)	253.3 (68.8)	0.001
LVMI (gr/m^2^)	106.2 (34.2)	82.3 (15.1)	133.4 (29)	0.001

Demographic parameters of patients with UCG-LVH vs controls. The models were two sided and significant p -value was <0.05

Abbreviations: SD: standard deviation LVH: left ventricular hypertrophy, BMI: body mass index, BSA: body surface area, IVSTd: interventricular septum thickness diastole, LVIDd: left ventricular internal diameter diastole, LVPWTd: left ventricular posterior wall thickness diastole, RWT: relative wall thickness, LVM: left ventricular mass, LVMI: left ventricular mass index.

Comorbidities in both groups are shown in **Table 2**. The number of patients with atrial fibrillation, chronic heart failure, hypertension, aortic stenosis and hypothyroidism were different between groups (*p<0*.*05*).

**Table 2:** Comorbidities of the population.

	Total sample* (n = 321, 100%)	Negative ECO-LVH (n = 145, 45.1%)	Positive ECO-LVH (n = 176, 54.8%)	p-value
AF	63 (19.6)	19 (13.1)	44 (25)	*0*.*011*
Aortic stenosis	17 (5.3)	1 (0.7)	16 (9.1)	*0*.*001*
IHD	171 (52.3)	84 (53.2)	87 (51.5)	*0*.*825*
CHF	44 (13.7)	13 (9)	31 (17.6)	*0*.*03*
CKD	32 (10)	9 (6.2)	23 (13.1)	*0*.*06*
COPD	9 (2.8)	1 (0.7)	8 (4.5)	*0*.*044*
Dyslipidemia	60 (18.7)	23 (15.9)	37 (21)	*0*.*253*
DM2	114 (35.5)	43 (29.7)	71 (40.3)	*0*.*061*
Hypertension	207 (64.4)	81 (25.2)	126 (39.2)	*0*.*001*
Hypothyroidism	33 (10.3)	9 (6.2)	24 (13.6)	*0*.*041*
OSA	3 (0.9)	1 (0.7)	2 (1.1)	*1*.*0*
PAD	15 (4.7)	8 (5.5)	7 (4)	*0*.*599*
PH	5 (1.6)	2 (1.4)	3 (1.7)	*1*.*0*
PE	7 (2.2)	4 (2.8)	3 (1.7)	*0*.*705*
SSS	3 (0.9)	1 (0.7)	2 (1.1)	*1*.*0*
Stroke	34 (10.6)	13 (9)	21 (11.9)	*0*.*467*
SVT	9 (2.8)	5 (3.4)	4 (2.3)	*0*.*736*

*Missing completely of random values of comorbidities were 25.7% of the total sample. Complete case analyses were performed.

Abbreviations: AF: atrial fibrillation, IHD: ischemic heart disease, CHF: congestive heart failure, CKD: chronic kidney disease, COPD: chronic obstructive pulmonary disease, DM2: type 2 diabetes mellitus, OSA: obstructive sleep apnea, PAD: peripheral artery disease, PH: pulmonary hypertension, PE: pulmonary embolism, SSS: sick sinus syndrome, SVT: supraventricular tachycardia.

**Fig 1:**
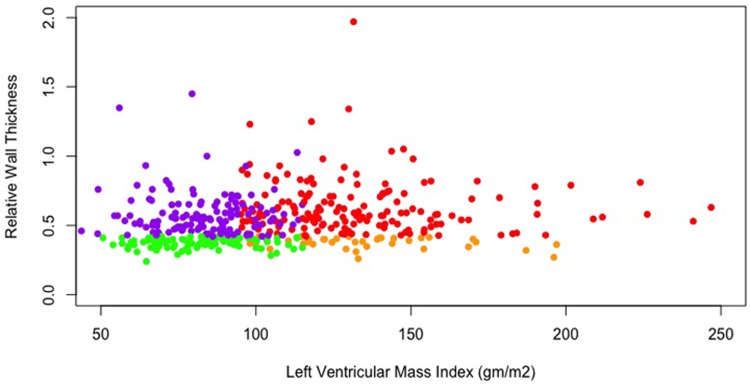
LVMI distributions and left ventricular morphologies. Morphologies of the left ventricle were defined as: normal (normal LVMI with RWT ≤0.42) (green), cardiac remodeling (normal LVMI with RWT >0.42) (purple), concentric LVH (elevated LVMI with RWT >0.42) (red) and eccentric LVH (elevated LVMI with RWT ≤0.42) (orange). LVH: left ventricular hypertrophy (male: >115 g/m^2^ and female: >95 g/m^2^), LVMI: left ventricular mass index, RWT: relative wall thickness.

As expected, all of the Echo measurements were statistically different between cases and controls (*p<0*.*01)* **(Table 1)**. Among all included patients, left ventricular morphology patterns were as follows: normal morphology (n = 100, 23.1%), cardiac remodeling (n = 130, 30.1%), concentric LVH (n = 165, 38.2%) and eccentric LVH (n = 37, 8.6%). LVH severity stage was classified as: mild (n = 77, 38.1%), moderate (n = 50, 24.7%) and severe (n = 75, 37.1%). There was no difference in the severity stage of LVH between males and females (*p = 0*.*420*). The distribution of LVMI, RWT and different left ventricle morphologies are shown in **Fig 1**.

### New criteria

The best logistic regression model (*AIC* = 524.8) is shown in **Table 3**. The presence of multiple variables pertaining to the right side of the heart, such as R_aVR and S_aVR is specified in the model.

**Table 3:** Logistic regression model.

	Estimate	Std Error	p-value	CI 95%
Intecept	-2.7	0.86	*0*.*001*	[0.01, 0.34]
ST abnormalities	1.28	0.30	*<0*.*001*	[1.98, 6.52]
S V4	0.93	0.36	*0*.*01*	[1.24, 5.26]
Intrinsicoid deflection in V6	.97	.32	*0*.*002*	[1.4, 5.04]
Negative P-wave deflection in V1	0.96	0.24	*<0*.*001*	[1.61, 4.22]
R aVR	3.9	1.3	*0*.*002*	[4.1, 685.1]
S aVR	0.48	0.34	*0*.*18*	[0.83, 3.17]
P-wave duration in V1	-0.009	0.003	*0*.*14*	[0.98, 0.99]
S V6	1.91	0.95	*0*.*04*	[1.05, 44]
S I	-2.24	1.01	*0*.*027*	[0.01, 0.77]
QRS duration	0.01	0.008	*0*.*1*	[0.99, 1.03]
R I	0.53	0.37	*0*.*15*	[0.81, 3.57]

AIC value: 524.8. These variables were used in the decision tree model. The estimates are standardized. The model was two sided and significant p -value was <0.05

Abbreviations: Std error: standard error, S V4: S-wave voltage in V4 lead, V6 intrinsicoid deflection: defined as a qR duration ≥ 0.05, negative P-wave deflection in V1 lead: defined as having a negative component duration greater than the duration of the positive component, R aVR: R-wave voltage in aVR lead, S aVR: S-wave voltage in aVR lead, S V6: S-wave voltage in V6 lead, S I: S-wave voltage in I lead, QRS duration: duration of QRS complex in V1 lead, R_I: R-wave voltage in I lead.

**Fig 2:**
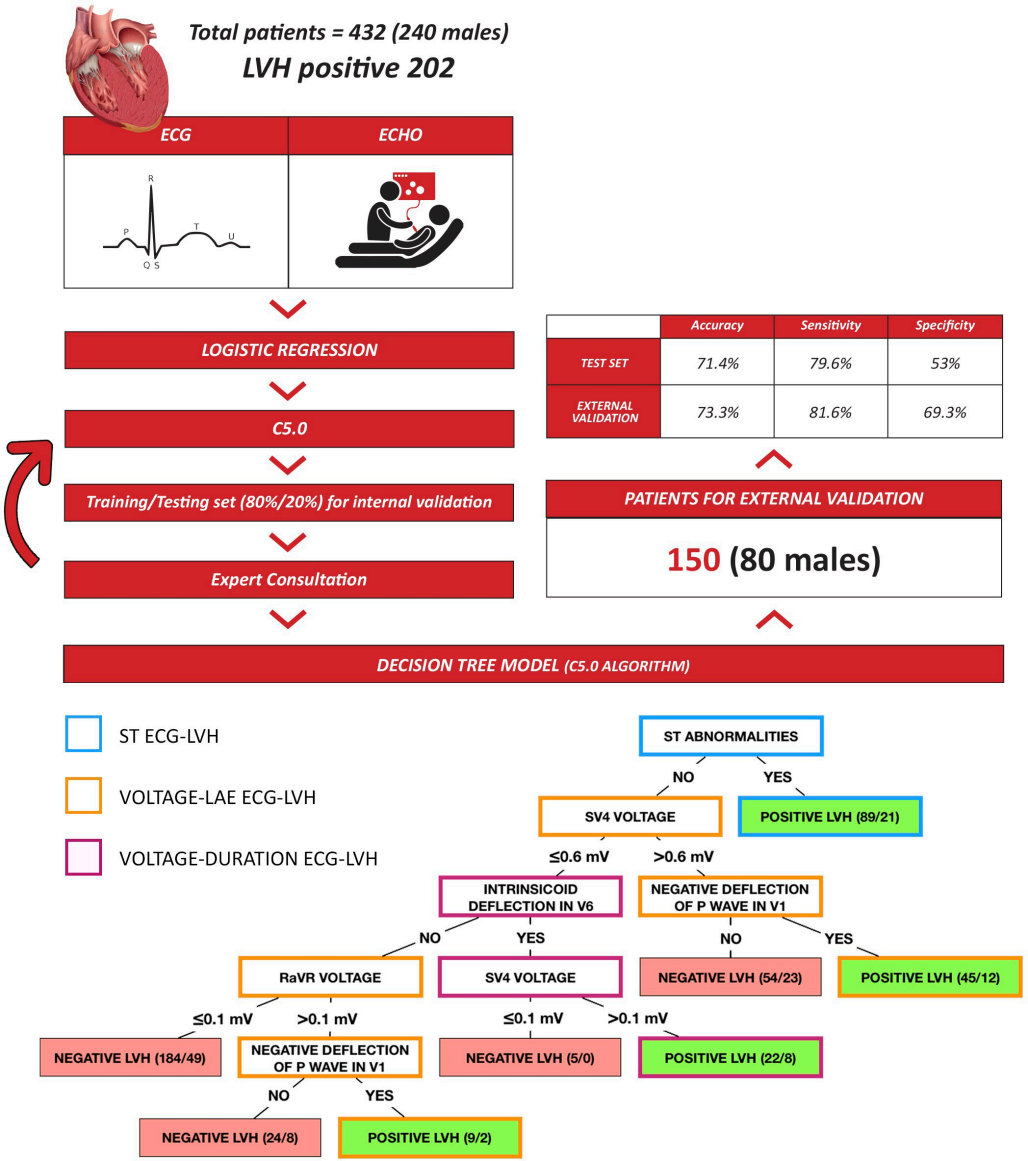
New model and electrocardiographic left ventricular hypertrophy phenotypes. Echo and ECG were obtained in 432 patients (46.7% LVH positive). Logistic regression modeling was performed for dimensionality reduction. This supervised classification model was created with 80% of the sample and the remaining 20% was used for internal validation. The *C5*.*0* ML algorithm resulted in a simple, five-level, seven-node decision tree. Each leaf shows the probability of having LVH and if greater than 0.5 (50%), the patient will be classified as having LVH and *vice-versa*. External validation was conducted in 150 subjects (47.3% LVH positive). ECG: Electrocardiography, ECHO: echocardiography, LVH: left ventricular hypertrophy.

The variables from the logistic regression model were used for the final decision tree. The performance with internal validation reached a diagnostic accuracy of 71.4%, (95%CI, 65.5–80.2), a sensitivity of 79.6%, specificity of 53%, PPV of 66.6%, and NPV of 69.3%. This model included only six predictive variables and had a size of seven nodes and 5 levels. Our new model and ECG-LVH phenotypes are presented in **Fig 2.**

#### External validation cohort

A cohort of seventy-one patients with Echo-LVH and seventy-nine controls was used for external validation. Eighty (53.3%) males were included, with a mean age of 64.4 years (13.9), and the overall BMI was 28.3 kg/m^2^ (5.0). Age (95%CI, -6.6–2.3; p = 0.348) and gender (p = 0.166) were similar in both groups. The diagnostic accuracy obtained in the external validation cohort was 73.3% (95%CI, 65.5–80.2). Sensitivity, specificity, PPV and NPV were 81.6%, 69.3%, 56.3% and 88.6%, respectively (See Fig 2).

#### Performance of different parameters of our decision tree

In order to evaluate if the first three ECG criteria of our decision tree performed good enough to diagnose LVH or if all the ECG parameters of the final model were needed, we tested four ECG combinations of these parameters. These combinations were also selected based in clinician expertise. Their diagnostic performances are shown in **Table 4**. The accuracy ranged between 55.3%-60.4%, which means that for better classification all parameters of our decision tree must be used.

**Table 4:** Diagnostic performance of simplified decision trees.

	Accuracy	Sensitivity (%)	Specificity (%)	PPV (%)	NPV (%)
SV4 AND ST abnormality	60.4	82.9	57.6	19.3	96.5
SV4 AND negative deflection in V1	60.2	75	57.8	22.3	93.5
Negative deflection in V1 AND ST abnormality	60.4	82.9	57.6	19.3	96.5
Negative deflection in V1 AND ST abnormality AND SV4	55.3	80	54.4	5.9	98.7

Abbreviations: PPV: positive predictive value, NPV: negative predictive value, SV4: positive if S-wave equal or greater than 0.6mV, major ST abnormality: defined as Minnesota’s code 4–1, Negative deflection in V1: P wave negative’s component duration in V1 lead is greater than the initial positive component.

### Romhilt-Estes multilevel score

The Romhilt-Estes multilevel score had an accuracy of 61.3% (95%CI, 56.5–65.9), a sensitivity of 23.2%, specificity of 94.8%, PPV of 79.6%, and NPV of 58.4%. In the external validation group, results were similar: 57.4% (95%CI, 49–65.5), 11.6%, 97.4%, 80%, 55.8%, respectively.; p = 0.3486.5ecimiento auricular)he duration of thede HVI-ECG en nuestra pobacidiferentes (ej. ST-strain y crecimiento auricular)

## Discussion

### Clinical and research implications

We demonstrated that the ML *C5*.*0* algorithm optimized the ECG to detect Echo-LVH by creating a simple and easy to use binary decision tree with seven nodes, five levels and six predictive variables that reflected three distinct ECG phenotypes **(Fig 2)**.

The model surpassed the current validated criteria (i.e. Romhilt-Estes, Cornell and Sokolov) [1], with an accuracy of 71.4%, (95%CI, 65.5–80.2). Our findings were validated in an external cohort, reaching a similar diagnostic accuracy. Also, we created four simplified decision trees with high applicability and a similar diagnostic performance to the current validated criteria (**Table 4**).

Historically, many authors have tried to improve ECG capabilities to detect LVH, by computing different ECG measurements and applying different statistical techniques [1, 15, 16]. The main problems with these approaches have been: 1) sensitivity and specificity mismatch (i.e. Romhilt-Estes) or 2) exclusion of ECG abnormalities with prognostic significance (i.e. Cornell or Sokolov criteria) [2, 3, 6, 16]. Our approach corrected both problems because the ML *C5*.*0* algorithm used highly relevant ECG features and provided appropriate cut-off values. The intrinsic characteristics of our model resulted in a highly interpretative, easy-to-trace path and included variables with prognostic value. It also provided insights on the electrogenesis of the hypertrophied myocardium [17].

Other advantages of our model were that it did not require patient information (i.e. digoxin usage in Romhilt-Estes or gender in Cornell) [5, 18] and it was easy to automate, thus decreasing operator bias [19].

Ventricular repolarization (ST-abnormalities) is of great relevance in the identification of Echo-LVH in terms of increased voltage. One must be cautious with isolated changes in voltage or depolarization time, and should not be used as an equivalent of Echo-LVH.

The Romhilt-Estes multilevel score components have shown prognostic implications in prospective studies [20]; however, its diagnostic accuracy was low in our population. This could be related to items that were not appropriately weighed (i.e. ST-strain pattern and LAE have the same score) and a lower prevalence of some of its components in our population (i.e. ST-strain after the hypertensive era) [5, 13].

### Model description

#### Major ST abnormalities

Ventricular repolarization represented the most important node in our model because it provided most of the information to classify Echo-LVH when identifying high-risk patients [21, 22]. The hallmarks of myocardial electrical remodeling are well-documented alterations in genes encoding Ca^2+^-handling proteins and inward L-type Ca^2+^ current channels, which further support our findings [23].

Although highly specific, the ST “strain” pattern is rare in our population so the decision tree did not include this abnormality [13]. The algorithm included another major ST-abnormality, Minnesota’s code 4–1 (MC 4–1), which has been associated with poor cardiovascular outcomes independently of the presence of coronary heart disease [21, 22, 24].

In order to decrease selection bias, we excluded several causes of ST depression (i.e. tachycardia) and included patients with a broad spectrum of ischemic heart disease. Nonetheless, it is important to exclude other obvious causes of MC 4–1 ST-abnormalities in order to apply our criteria. False dichotomy has been reported with other criteria (i.e. Cornell), exemplified by cases in which if a supposed voltage threshold is surpassed, the patient is classified as having LVH [1].

ST-abnormalities can be found in patients with conditions associated to pressure overload (i.e. aortic stenosis and arterial hypertension). These conditions were more commonly found in the Echo-LVH group but ST-abnormalities were no different in patients with or without these conditions.

#### Voltage and conduction delay

Increased QRS voltage and conduction delay are well-documented manifestations in the hypertrophied myocardium and are the most common ECG abnormalities used to detect Echo-LVH (i.e. Cornell, Romhilt-Estes); both have been associated with poor cardiovascular outcomes [1, 25].

Many factors influence voltage and duration such as patient characteristics (age, gender, race, body habitus), spatial parameters (distance of recording lead) and non-spatial parameters (intra and extracellular conductivity). These could be related to variability in accuracy (0–50%) and sensitivity (<30%) [1].

We believe that voltage and duration should be used only in conjunction with other types of criteria. In our model, voltage classified patients as having Echo-LVH only if there also was conduction delay or LAE but never solely on voltage **(Fig 2).** In a small cohort study, R-wave voltage in the aVR lead was reported to be useful to classify patients with Echo-LVH [26]; in our algorithm, the aVR lead voltage also helped to classify these patients **(Fig 2).**

#### Left atrial enlargement

Hypertension is one of the most common etiologies of LVH and LAE, and represents an early ECG finding in hypertensive cardiopathy [1]. Classically, LAE is defined as a negative P terminal force equal or greater than one Ashman unit using the V1 lead (i.e. Romhilt-Estes) and although highly specific, it has low sensitivity (≈12%) [5, 27]. Therefore, we decided to include another ECG definition of LAE (see Method section) that was found in two different positions, to classify patients with Echo-LVH **(Fig 2)**; This could represent a subgroup of patients with Echo-LVH and no ventricular ECG findings, but it requires further exploration. In patients with atrial fibrillation, a condition commonly associated with LVH, this node will become falsely negative and requires further investigation.

We used LAE criteria in conjunction with other type of ECG abnormalities in order to diagnose ECG-LVH [1].

### Limitations and future research

This study is focused in identifying and understanding the relationship between mechanic (Echo-LVH), bioelectrical LVH (ECG-LVH) characteristics and their interrelations with clinical outcomes according to the recommendations of the Working Group on Electrocardiographic Diagnosis of Left Ventricular Hypertrophy [17, 28]. We recognize that ECG-LVH predicts cardiovascular events independently of the ventricular mass, indicating that Echo-LVH and ECG-LVH are different but somehow connected processes [29].

The ECG requires further optimization for morphological analysis of the heart. More accurate and complex ECG measurements are needed in order to conclude that an ECG is a low accuracy tool when attempting to predict LVH. We believe that the most important limitation of the ECG in performing this task is human dependency. The clinician is limited to certain ECG measurements (i.e. voltage and duration), but omits others that are relevant (i.e. areas under the curve, ST slope, QRS area). Increasing the quality of the data input in the *C5*.*0* ML algorithm or other ML algorithms seems mandatory in order to create a powerful tool to detect LVH.

## Conclusions

In conclusion, the *C5*.*0* ML algorithm surpassed the accuracy of the currently used ECG criteria to detect Echo-LVH in our population. These criteria can be used in specific populations that are very common in our population. Our new criteria hinge on ECG abnormalities that identify high-risk patients and provide insight on electrogenesis in Echo-LVH. In the field of electrical morphology analysis of the heart, it is paramount to conserve the ability of the clinician to interpret results; to achieve this we used a non-black box artificial intelligence algorithm so that the specific electrical alteration associated to Echo-LVH can be easily visible. Furthermore, this model is simple and can be easily understood by any healthcare member, so we think that this algorithm will be very useful in the physician daily practice.

## Supporting information

S1 Dataset Data used for training and internal validation of the model. (XLS)

S2 Dataset (XLSX)
